# Use of Norovirus Genotype Profiles to Differentiate Origins of Foodborne Outbreaks

**DOI:** 10.3201/eid1604.090723

**Published:** 2010-04

**Authors:** Linda Verhoef, Harry Vennema, Wilfrid van Pelt, David Lees, Hendriek Boshuizen, Kathleen Henshilwood, Marion Koopmans, on behalf of the Food-Borne Viruses in Europe Network

**Affiliations:** National Institute for Public Health and the Environment, Bilthoven, the Netherlands (L. Verhoef, H. Vennema, W. van Pelt, H. Boshuizen, M. Koopmans); Centre for Environment, Fisheries and Aquaculture Science, Weymouth, UK (D. Lees, K. Henshilwood)

**Keywords:** Norovirus, genotype, food, infectious diseases, transmission, surveillance, outbreaks, viruses, epidemiology, correlation, research

## Abstract

Because secondary transmission masks the connection between sources and outbreaks, estimating the proportion of foodborne norovirus infections is difficult. We studied whether norovirus genotype frequency distributions (genotype profiles) can enhance detection of the sources of foodborne outbreaks. Control measures differ substantially; therefore, differentiating this transmission mode from person-borne or food handler–borne outbreaks is of public health interest. Comparison of bivalve mollusks collected during monitoring (n = 295) and outbreak surveillance strains (n = 2,858) showed 2 distinguishable genotype profiles in 1) human feces and 2) source-contaminated food and bivalve mollusks; genotypes I.2 and I.4 were more frequently detected in foodborne outbreaks. Overall, ≈21% of all outbreaks were foodborne; further analysis showed that 25% of the outbreaks reported as food handler–associated were probably caused by source contamination of the food.

## Abstract 2

Detection should enable containment of viral foodborne infections.

Noroviruses are members of the family *Caliciviridae* and recognized as major pathogens in outbreaks of gastroenteritis worldwide. Because these viruses have environmental stability (*1*), ability to use different transmission routes, and low infective doses (*2*), their source may be difficult to determine during an outbreak. Transmission can occur through contact with shedding persons; food contaminated during processing, preparation or serving; sewage-contaminated water used for consumption, cultivation or irrigation of food; contaminated aerosols resulting from vomiting; and environmental contamination (*3*,*4*). Five genogroups have been described (GI–V), subdivided into at least 40 genetic clusters (*5*,*6*).

To implement effective measures for prevention, recognition of the transmission routes is necessary. Consequently, the relative importance of different transmission routes in the total number of outbreaks is of interest for estimation of cost-effectiveness of reducing the number and size of norovirus outbreaks, particularly for geographically disseminated foodborne outbreaks. Such outbreaks are difficult to detect when the primary introduction of viruses through food occurs simultaneously in several countries or continents (*7*–*9*). Globalization of the food industry with consequential international distribution of products increases the risk for such outbreaks. For example, the first reported GII.b outbreak occurred in August 2000 during a large waterborne outbreak in southern France (*10*). After this outbreak, in December and January, 4 multipathogen and oyster-related outbreaks with this newly emerging genotype were reported from France. In the same period, Denmark, Finland, and the Netherlands reported norovirus cases resulting from oysters originating from a French batch that probably was sold in these countries, as well as in Sweden, Italy, and Belgium (*6*). All these outbreaks seemed to involve closely related and newly detected GII.b strains. After active case identification, further linked cases were detected in Germany, the United Kingdom, Spain, Slovenia, and Sweden (*11*,*12*). Another example of a geographically disseminated outbreak was several seemingly independent norovirus outbreaks in Denmark that were traced back to consumption of raspberries from Poland. Although raspberries from this contaminated batch were exported to other European countries, an alert in the Rapid Alert System for Food and Feed did not result in further linked outbreak reports (*7*). Thus, geographically disseminated outbreaks are sometimes identified but only after the joint and exhaustive efforts of different organizations, such as laboratory networks, food safety authorities, and public health institutions. Knowledge of the proportion of geographically disseminated foodborne outbreaks to all norovirus outbreaks will therefore provide insight into the cost-effectiveness of such efforts.

We studied whether the genotype frequency distributions (genotype profiles) of strains can be used to differentiate foodborne outbreaks related to contamination early in the food chain (i.e., during primary production) from those related to contamination later in the food chain (i.e., during preparation or serving). If so, detection of food origins likely to cause geographically disseminated outbreaks will be enhanced. We considered methods for attribution to multiple sources commonly applied to *Salmonella* infections (*13*) because different transmission routes involved in norovirus infections can disguise the foodborne origin. However, such methods require strain collections representative of noroviruses in the potential sources that are as yet unavailable because of difficulties in the direct detection of viruses in food (*14*–*16*). Therefore, we compared 2 strain collections: noroviruses identified through filter-feeding bivalve mollusk monitoring representing source contamination of food and noroviruses collected through systematic surveillance of illness in the population. The first was collected by the European Community Reference Laboratory for Monitoring Bacteriological and Viral Contamination of Bivalve Mollusks during 1995–2004 (*17*) and the second by the Food-Borne Viruses in Europe (FBVE) network, which has conducted surveillance for norovirus outbreaks in Europe since 1999. Prior investigation of the FBVE database of systematically collected epidemiologic and microbiological norovirus surveillance data (*6*) showed that the epidemiology of norovirus outbreaks in Europe varies between genogroups. An analysis of the properties of reported outbreaks indicated a clear difference between GII.4 strains and other noroviruses; non-GII.4 strains were found more frequently in outbreaks with a foodborne mode of transmission, and GII.4 strains were found more frequently in healthcare settings with person-to-person transmission (*18*,*19*). Here we demonstrate that further specification into genotypes shows additional differences in the epidemiology of norovirus outbreaks.

## Methods

### Data Sources

We used 2 broad databases reflecting norovirus prevalence within the European countries under surveillance. These databases provided us the opportunity to compare genotype proportions as detected in outbreaks, i.e., human surveillance data, with those detected in source-contaminated food products, i.e., bivalve mollusks monitoring data.

#### Human Surveillance Data

From January 1999 through December 2004, FBVE collected molecular information on 2,727 norovirus outbreaks and sporadic cases in Denmark, Finland, France, Germany, England and Wales, Hungary, Ireland, Italy, the Netherlands, Norway, Sweden, Slovenia, and Spain (*20*,*21*). Although the name FBVE suggested a foodborne focus, the network actually investigated outbreaks from all modes of transmission to obtain a comprehensive overview of viral activity in the community (strengths and limitations of the FBVE data collection were described by Kroneman et al. [*20*]; to compare newly detected strains with the FBVE database and find potential linked outbreaks, we used a comparison tool [www.rivm.nl/bnwww]). Data were reported to FBVE at outbreak level; therefore, no informed consent was needed. Outbreaks were categorized as follows on the basis of the cause of infection as reported in the surveillance system:

- Foodborne-food (FB-food) when an outbreak was reported to be caused by food and the outbreak strain was detected in food;
- Foodborne-feces (FB-feces) when an outbreak was reported to be caused by food and the outbreak strain was detected in human feces only;
- Foodborne (FB) when an outbreak was classified as FB-food or FB-feces;
- Food handler–borne (FHB) when an outbreak was reported to be caused by an infected food handler contaminating the food and the outbreak strain was detected in human feces;
- Person-borne (PB) when an outbreak was reported to be caused by person-to-person transmission and the outbreak strain was detected in human feces;
- Unknown (UN) when the mode of transmission was not reported or was reported to be unknown and the outbreak strain was detected in human feces.

When the mode of transmission was not reported but information was given in text data fields, this information was used to categorize the outbreak. Because we were interested in the origin of the virus, we categorized outbreaks involving PB transmission but starting with food as FB-food, FB-feces, or FHB, depending on available information. Strains detected in sporadic cases were clustered into outbreaks if information was available. The remaining strains detected in sporadic cases were considered of interest with respect to the genotypes causing human illness and representative of potential unreported outbreaks. When we detected multiple genotypes during an outbreak or in sporadic cases, we recorded each genotype.

#### Bivalve Mollusk Monitoring Data

The European Community Reference Laboratory for Monitoring Bacteriological and Viral Contamination of Bivalve Mollusks systematically collected sequence data on norovirus strains routinely detected in bivalve mollusks in Europe. During January 1999–December 2004, the laboratory systematically collected 295 strain sequences with region A sequence lengths varying from 76 to 78 nt. These strain sequences were detected as part of production area monitoring studies or outbreak investigations of gastroenteritis in Denmark, England and Wales, Ireland, Scotland, and Spain. All samples were first routinely tested with GI and GII PCR methods; then all positive samples were cloned (*22*), resulting in a representative reflection of norovirus presence in bivalve shellfish. If we detected multiple genotypes in 1 sample, we recorded each genotype.

### Assignment of Genotypes

Strains were genotyped by using a previously described method for sequence analysis of a fragment of the RNA-dependent RNA polymerase gene regions B, C, and D (*23*) because these regions were used in the FBVE network. From the start, the network used sequence-based genotyping of the then most commonly used diagnostic PCR fragment, targeting the RNA-dependent RNA polymerase gene. Since then, however, it has become clear that recombination is common and mainly occurs in the area between the overlap between the polymerase and the capsid gene. Therefore, capsid-based and polymerase-based typing may be discordant. Genotype assignment was therefore performed only after clustering of query strains against all relevant available sequences in the FBVE database (M. Koopmans et al., unpub. data). This process resulted in the genotyping of all but 68 (2%) strains. Genotypes were classified on the basis of their similarity to reference strains representing known genotypes by using the norovirus typing library (www.noronet.nl/nov_quicktyping). If the (clustered) genotypes occurred <5 times in our 5-year covering data selection, the frequencies were considered too low to be ascribed a separate genotype and excluded. This was the situation for GII.18 and 6 clusters of nonassigned GII strains (n = 25, 1%).

### Data Analysis

First, we compared the genotype frequency distributions detected in outbreak categories reported as FB-food, FB-feces, FHB, PB, and UN and in routinely tested bivalve mollusks. To evaluate the correlation and measures of association of these 6 proportional profiles, Pearson correlation coefficient ρ was calculated on the basis of frequencies (ρ_1_) and logarithm (ρ_2_) of the frequencies of 22 genotypes, as well as Cramer V and simulated p values by using 20,000 replications with the exact variant of the χ^2^ test. The exploratory technique correspondence analysis allows for examining the structure of categorical variables in a multiway table and was used to visualize the measure of correspondence in the 6 genotype profiles. p values <0.05 were considered significant.

Second, to differentiate the remaining genotype profiles detected in outbreaks, we used the genotype profiles of the 2 main transmission modes to be distinguished during an outbreak investigation. For each genotype in the human surveillance collection, the fraction of outbreaks of known origin being FB (i.e., FB-food and FB-feces) or PB was estimated on the basis of the proportion of FB outbreaks of all FB + PB outbreaks in each genotype. We used the estimated proportion of FB outbreaks of all FB + PB outbreaks in each genotype to estimate the probability that an FHB or UN outbreak was foodborne. We calculated 95% confidence intervals (CIs) using Monte Carlo simulation with 10,000 random draws from the β distributions, which are the posterior probabilities of the proportions (*24*).

## Results

Of 3,022 detected noroviruses, 25 (1%) were excluded because of low frequencies; for 68 (2%), assignment of a genotype was not possible because of short sequences or inability of the method applied to type the detected norovirus beyond its genogroup. Of the remaining 2,929 strains, 71 (2%) could not be linked to epidemiologic data, and therefore their origin remained unknown, leaving 2,858 (95%) strains for analysis: 922 originating from PB outbreaks, 24 from FB-food outbreaks, 151 FB-feces outbreaks, 20 FHB outbreaks, 1,446 UN outbreaks, and all 295 bivalve mollusk monitoring strains. Among the outbreaks of known origin, 175 (16%) of 1,117 were reported to be FB (i.e., FB-food and FB-feces).

The proportion of genogroup I was significantly higher in bivalve mollusks (137/295, 46%) than in infected humans (313/2,539, 12%) (Table 1). All genotypes detected in bivalve mollusks were also detected in humans; however, 9 genotypes causing human illness were not detected in bivalve mollusks. Overall, the II.4 genotype was responsible for most of the human outbreaks (1,326/2,539, 52%), followed by II.b (328/2,539, 13%) and II.7 (156/2,539, 6%).

**Table 1:** Number of norovirus strains detected in samples from humans, bivalve mollusks, and food, 1999–2004*

Genotypes			Human surveillance, no. strains					Bivalve monitoring, no. strains	Total no. strains
Pol-based	Cap-based		FB-food	FB-feces	FHB	PB	UN		
Genogroups									
I.1	I.1		1	8	0	5	18	0	32
I.2			0	6	0	1	32	8	47
I.3	I.3		0	8	3	16	80	13	120
I.4	I.4		9	8	1	8	46	86	158
I.5			0	0	0	1	5	3	9
I.6	I.6		2	3	1	21	17	25	69
I.7			0	1	0	0	7	2	10
NA I.a	NA I.a		0	1	0	0	4	0	5
II.1			0	5	2	12	94	7	120
II.2	II.2		0	13	1	27	66	0	107
II.3			0	1	0	1	38	11	51
II.3R	II.3		0	1	0	1	41	2	45
II.4	II.4		5	47	9	681	584	63	1,389
II.5			0	3	0	6	12	0	21
II.8			1	0	1	1	13	0	16
NA II.a			0	0	0	2	7	0	9
NA II.c			0	2	0	8	31	1	42
NA II.d			0	1	0	3	8	0	12
IV.1			0	2	0	1	8	0	11
Recombinants									
NA II.b	II.1, II.2, II.3		4	23	1	100	200	63	391
II.1	II.10		0	0	0	8	19	11	38
II.7	II.6, II.7		2	18	1	19	116	0	156
Total			24	151	20	922	1,446	295	2,858

*Poly, polymerase; cap, capsid; FB-food, foodborne-food, i.e., an outbreak was reported to be caused by food and the outbreak strain was detected in food; FB-feces, foodborne-feces, i.e., an outbreak was reported to be caused by food and the outbreak strain was detected in human feces only; FHB, food handler–borne, i.e., an outbreak was reported to be caused by an infected food handler contaminating the food and the outbreak strain was detected in human feces; PB, person-borne, i.e., an outbreak was reported to be caused by person-to-person transmission and the outbreak strain was detected in human feces; UN, unknown, i.e., the mode of transmission was not reported or was reported to be unknown and the outbreak strain was detected in human feces.

**Figure A1:**
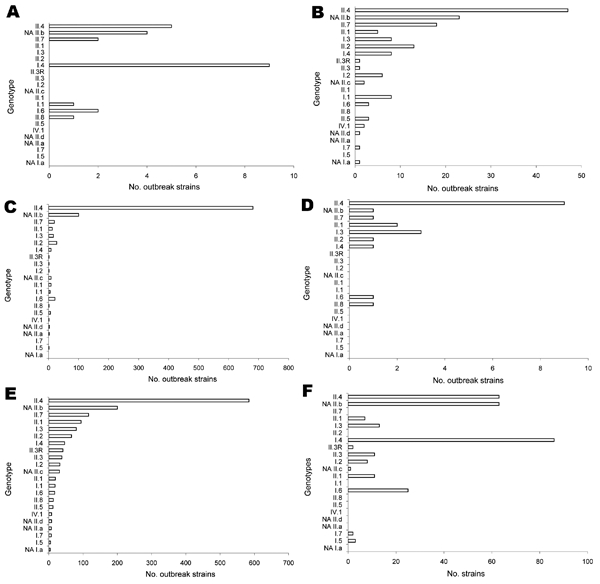
Norovirus genotype profiles detected in foodborne outbreaks, showing strains detected in A) food or B) humans; C) person-borne outbreaks; D) food handler–borne outbreaks; E) outbreaks with an unknown mode of transmission; and F) routine monitored bivalve mollusks. The genotypes are sorted for their relevance in unknown outbreaks.

We visualized genotype frequency distributions as profiles for the observed categories of outbreaks and sorted them for their relevance in UN outbreaks, presented with different scales allowing for proportional comparison (Figure A1). The genotype profiles vary between these groups. The correlation coefficients based on frequencies, ρ_1_, showed that 2 genotype profiles were distinguishable (Table 2): 1 profile typically seen in human feces (FB-feces, FHB, or PB), and another profile typically detected in sources other than human feces, i.e., in food (FB-food) or bivalve mollusks. The ρ_1_ reflects some genotypes frequently and others rarely seen in FB-food and bivalve mollusks. Because FB-food strains include oyster-related outbreaks as well, we assumed that the correlation between FB-food and bivalve mollusks can be explained partly by these oyster-related outbreaks. We therefore calculated an additional correlation coefficient using the 14 strains detected in food items other than bivalve mollusks. Despite low numbers, this calculation resulted in a high, significant correlation coefficient (ρ = 0.81, p <0.001). The logarithm of the frequencies, ρ_2_ (Table 2), is less sensitive to peak frequencies of genotypes and therefore capable of differentiating profiles with respect to the rare genotypes and approaching the Cramer V. Cramer V and ρ_2_ show less clear association of profiles, with diverging results for the FHB and UN profiles.

**Table 2:** ρ1, ρ2, and Cramer V results with simulated p values (20,000 replications) of norovirus 6 genotype patterns as detected in routinely tested bivalve shellfish and during norovirus outbreaks, 1999–-2004*

Source	FB-food (p value)	FB-feces (p value)	FHB (p value)	PB (p value)	UN (p value)	Bivalve mollusk (p value)
FB-food						
ρ_1_	1.00	0.48 (0.02)	0.40 (0.07)	0.43 (0.04)	0.48 (0.02)	0.91 (<0.01)
ρ_2_		0.46 (0.03)	−0.15 (0.51)	0.34 (0.12)	0.24 (0.26)	0.47 (0.03)
Cramer V		0.47 (0.01)	0.62 (0.04)	0.48 (<0.01)	0.26 (<0.01)	0.41 (0.02)
FB-feces						
ρ_1_		1.00	0.93 (<0.01)	0.92 (<0.01)	0.96 (<0.01)	0.53 (0.01)
ρ_2_			0.40 (0.06)	0.55 (<0.01)	0.66 (<0.01)	0.69 (<0.01)
Cramer V			0.34 (0.41)	0.43 (<0.01)	0.19 (<0.01)	0.57 (<0.01)
FHB						
ρ_1_			1.00	0.97 (<0.01)	0.93 (<0.01)	0.43 (<0.05)
ρ_2_				0.22 (0.32)	0.39 (0.07)	0.47 (0.03)
Cramer V				0.25 (<0.01)	0.09 (0.75)	0.46 (<0.01)
PB						
ρ_1_				1.00	0.96 (<0.01)	0.51 (0.01)
ρ_2_					0.61 (<0.01)	0.53 (0.01)
Cramer V					0.42 (<0.01)	0.63 (0.01)
UN						
ρ_1_					1.00	0.59 (<0.01)
ρ_2_						0.65 (<0.01)
Cramer V						0.48 (<0.01)
Bivalve mollusk						1.00

*ρ_1_ = based on frequencies; ρ_2_ = based on logarithm of frequencies; Cramer V, χ^2^ test with simulated p values; FB-food, foodborne-food, i.e., an outbreak was reported to be caused by food and the outbreak strain was detected in food; FB-feces, foodborne-feces, i.e., an outbreak was reported to be caused by food and the outbreak strain was detected in human feces only; FHB, food handler–borne, i.e., an outbreak was reported to be caused by an infected food handler contaminating the food and the outbreak strain was detected in human feces; PB, person-borne, i.e., an outbreak was reported to be caused by person-to-person transmission and the outbreak; strain was detected in human feces; UN, unknown, i.e., the mode of transmission was not reported or was reported to be unknown and the outbreak strain was detected in human feces.

Table 2 shows the quantification of association; the associated genotype profiles illustrated by correspondence analysis is shown in the Figure. The values of the 6 columns in Table 1 can be considered coordinates in a 6-dimensional space, and the distances are computed. These distances summarize information about the similarity between the rows in Table 1. Dimension 1 may be considered to differentiate transmission modes explaining 59.12% of the correspondence, confirming that the profiles found in bivalve mollusks and FB-food are similar with regard to the pattern of relative frequencies in genotypes (rows in Table 1) and differ from those in PB. It also shows that the FHB, UN, and FB-feces profiles are mutually similar, with their distance somewhere between the PB and FB-food/bivalve mollusk profiles. Dimension 2 may represent dual origin, explaining an additional 31.40%, showing that FB-feces, FHB, and UN mutually correspond and differ from FB-food, bivalve mollusks, and PB that mutually correspond.

**Figure A2:**
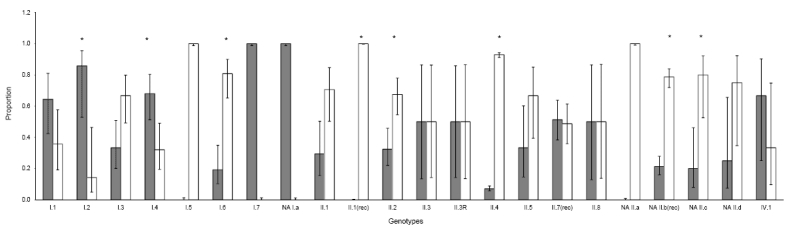
Estimated proportion of norovirus genotypes based on proportions as detected in foodborne outbreaks (i.e., detected in food or humans; gray bars) and person-borne outbreaks (white bars). *Indicates significance.

When we compared the proportions of genotypes detected in FB outbreaks with those in PB outbreaks, we detected genotypes I.2 and I.4 significantly more frequently in FB outbreaks (Figure A2). On the other hand, genotypes I.6, II.1W, II.2, II.4, II.b, II.c, and II.d were detected significantly more frequently in PB outbreaks. Using these proportional FB and PB genotype profiles and their confidence intervals to distinguish between FB and PB transmission among 20 FHB outbreaks, we could ascribe 5 (95% CI 4–6) to FB and 15 (95% CI 14–16) to PB transmission. Ascribing 1,446 unexplained human norovirus outbreaks to either FB or PB transmission resulted in ≈367 (95% CI 327–417) FB outbreaks and ≈1,079 (95% CI 1,026–1,120) PB outbreaks. Overall, use of the genotype patterns increases the estimated number of FB proportion of outbreaks to 21% (547/2,563; range 20%–23%) compared with the 16% previously mentioned among the outbreaks of known origin.

## Discussion

Our combined epidemiologic and virologic analysis demonstrated that norovirus genotype profiles, derived from long-term norovirus strain collections, can be used to differentiate foodborne outbreaks caused by food contamination early in the food chain from those caused by food handlers contaminating food. Our study is one step in deriving practical applicable information from the existing record and possible only through the availability of continuously updated databases containing detailed epidemiologic data and virus characterization. We confirmed a significant difference in the GI:GII ratio; GI strains were more prevalent in bivalve mollusks. On the basis of the 5-year strain collections, some genotypes (I.2 and I.4) suggest FB instead of PB preference, and others (II.2 and II.6/II.7) are commonly seen in outbreaks but not detected in bivalve mollusks (and FB-food). Strains detected in food that caused outbreaks (FB-food) showed a genotype profile similar to those in bivalve mollusk monitoring and dissimilar to the profile detected in human feces (i.e., FB-feces, FHB, PB, UN) with respect to the frequently seen genotypes. This finding may reflect the ability of these genotypes to survive outside humans or their diminished ability to spread or replicate within the human population. Genotype profiles of FHB and UN resulted in diverging association outcomes, which may reflect their potential dual origin.

Although consumption of contaminated food causes both types of outbreaks, outbreaks resulting from infected food handlers clearly necessitate different measures than do outbreaks resulting from food contaminated early in the food chain. Consequently, differentiation of these modes of transmission is of interest to food safety authorities and public health institutions. Food handler–borne outbreaks are end-of-chain outbreaks easily recognized as such, as numerous outbreak reports illustrate (*25*–*29*). Such outbreaks can be prevented or limited by exclusion of infected or shedding food handlers from work until 48–72 hours after recovery (*25*,*27*,*29*,*30*), education of food handlers (*26*), and standard testing of food handlers during outbreaks (*28*). A common source of contamination early in the food chain, however, may be more difficult to detect. Such contamination may result from sewage influx containing multiple viruses (*8*,*9*,*31*), making a link difficult to identify (*31*). Moreover, sewage most likely contains noroviruses from person-to-person outbreaks, which can contaminate the food and thereby dilute the genotype profiling effect. Use of genotype profiles is a first step toward recognizing outbreaks resulting from contamination early in the food chain because it allows estimation of the incidence in surveillance data retrospectively and objectively minimizes misclassification of outbreaks. However, genotyping data need to be interpreted with care, and continuous updating of the database remains necessary.

Our study has some limitations. First, our measures of association could not detect differences between genotype profiles with respect to the rare genotypes. Even so, the rare outbreak or sporadic strains are of interest because they may represent potential emerging or zoonotic genotypes with consequences for public health. Types that were initially rare may remain in human surveillance, as seen with the emergence of GII.b after a large waterborne outbreak (*10*) followed by, among others, foodborne distribution throughout Europe. Since then, GII.b strains have caused 13% of all outbreaks (Table 1), now mainly PB, suggesting good adaptation. On the other hand, if the rare types are unable to adapt for persistence in the human population, they may be repeatedly reintroduced, causing only sporadic cases but not outbreaks. This repeated introduction of sporadic cases would remain undetected at present because routine surveillance for sporadic cases is rare (*32*) and is not the current practice of FBVE. To identify the origin of newly emerging and rare strains, systematic monitoring of additional potential sources, such as cattle and swine (*33*) as well as sporadic human cases, is necessary.

Second, in our analysis, the transmission route was reported as unknown for 57% of outbreak strains. Incompleteness of surveillance data is a common problem (*34*) and has been recognized in surveillance of foodborne viral infections (*35*), including in the FBVE database (*19*,*20*). Incomplete data may have resulted in underestimation of the number of foodborne outbreaks because they may be complicated to identify. Food safety authorities routinely confirm FB clusters by detecting pathogens in food, but such confirmation is difficult for viruses because viruses, unlike bacteria, do not replicate in food, resulting in a low viral load for extraction and concentration. In addition, the matrix involved may complicate these procedures, and successful detection methods are available primarily for fresh produce with surface contamination and virus-accumulating shellfish (*36*,*37*). However, knowledge of the prevalence of strains in the environment, foods, and humans is necessary for the interpretation of matching. Such knowledge requires monitoring, which is limited to shellfish and norovirus outbreaks (*38*). For monitoring of foods other than shellfish, methods sensitive enough to detect viruses in naturally contaminated (and not spiked) food are required. The technical advisory group (TAG 4) of the Viruses in Food workgroup (WG 6) in the Technical Committee of Horizontal Methods for Food Analysis (TC 275) of the European Committee for Standardization (CEN) is validating standard methods for norovirus detection in bivalve mollusks, soft fruit, leafy vegetables, and bottled water (*39*). Until such methods are available and provide knowledge about the prevalence of viral presence in foods, the use of genetic profiles retrospectively derived from outbreak surveillance data is likely to improve foodborne viral surveillance. Because the norovirus strain population is continuously evolving, our analysis needs to be repeated periodically to ensure that retrospective findings remain predictive.

Third, international comparison of norovirus strains is complicated because of their genetic diversity and the involvement of several laboratories in diagnosis; consequential different assays result in sequences with diverging lengths and from diverging genomic regions. However, this limitation is not likely to have influenced our results because it affects mostly the comparison of sequence clusters and not genotypes. Moreover, within FBVE, standardization of diagnostic methods occurs by having participating laboratories regularly test a representative panel of fecal samples (*40*).

We showed that norovirus genotype profiles can be used to estimate the foodborne proportion of norovirus outbreaks while excluding those of the food handler as a source. Distinction at genogroup level had already indicated epidemiologic differences (*19*), and we have now demonstrated that genotype profiles can be used to differentiate transmission modes. The profiles and proportions are likely to be helpful for estimating the number of outbreaks with potential of causing geographically disseminated outbreaks. Because identification and investigation of such outbreaks provides insight into effective prevention measures during the production process, detection should enable containment of viral foodborne infection and thus prevent further spread and the consequent potential for large numbers of human infections.

## Figures and Tables

**Figure:**
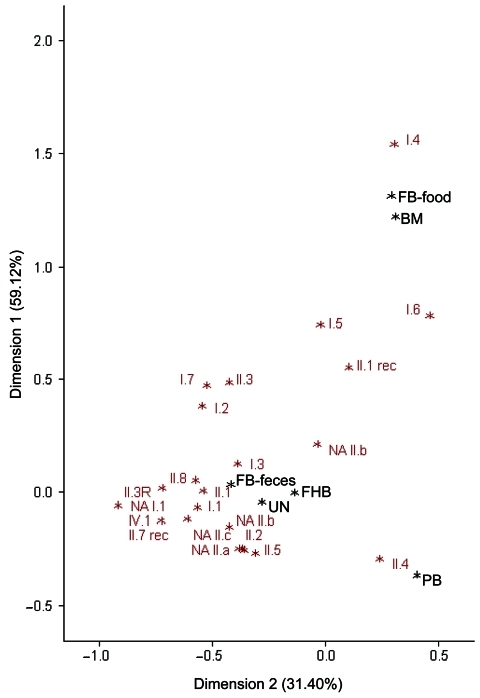
Two-dimensional display of the correspondence analysis of 6 norovirus genotype profiles based on nucleotide sequences in which points close to each other are similar with regard to the pattern of relative frequencies across genotypes. Dimension 1 explains 59.12% and dimension 2 an additional 31.40%. In dimension 1, foodborne-feces (FB-feces; i.e., outbreak reported to be caused by food with the outbreak strain detected in human feces only) and bivalve mollusk (BM) genotype profiles are mutually similar and differ from other profiles; the most distinct profile is person-borne (PB; i.e., an outbreak reported to be caused by person-to-person transmission with the outbreak strain detected in human feces). In dimension 2, food handler–borne (FHB; i.e., outbreak reported to be caused by an infected food handler contaminating the food with the outbreak strain detected in human feces), FB-feces, and unknown (UN; i.e., mode of transmission was not reported or was reported to be unknown with the outbreak strain detected in human feces) mutually correspond and differ from the mutually corresponding foodborne-food (FB-food; i.e., outbreak reported to be caused by food with the outbreak strain detected in food), BM, and PB.
